# Supplementary material for: Use, Practices and Attitudes of Sports Nutrition and Strength and Conditioning Practitioners towards Tart Cherry Supplementation
Source: Sports (Basel). 2020 Dec 22;9(1):2. doi: 10.3390/sports9010002 (PMC7821940; doi:10.3390/sports9010002)
Supplement: Supplementary file 1 [file sports-09-00002-s001.pdf]

sports

Supplementary Materials

Use, practices and attitudes of sports nutrition and strength and conditioning practitioners towards tart cherry supplementation

Vlad Sabou [1], Jimmy Wangdi [1,2], Mary F. O'Leary [1], Vincent G. Kelly [3] and Joanna L. Bowtell [1,*]

[1] Sport and Health Sciences, University of Exeter, St Luke's Campus, Heavitree Road, EX1 2LU Exeter, UK; vs348@exeter.ac.uk (V.S.); M.OLeary@exeter.ac.uk (M.F.O.)
[2] School of Human Movement and Nutrition, University of Queensland, Brisbane QLD 4072, Australia; j.wangdi@uq.net.au
[3] School of Exercise and Nutrition Sciences, Queensland University of Technology, Brisbane QLD 4000, Australia; v6.kelly@qut.edu.au
* Correspondence: J.Bowtell@exeter.ac.uk

The use and attitudes of sports nutrition and strength & conditioning practitioners towards tart cherry supplementation

Integral version of the questionnaire.

Section 1: Demographic-related questions/statements

1. Please indicate your age.

2. Please indicate your gender.

3. Please indicate from the list below your support role.

a) Sports nutritionist / dietician
b) Strength and conditioning coach
c) Athletic performance coach
d) Other:

4. Please indicate all the sports in which you are professionally involved (as self-employed or/ part-time/ full-time employed).

a) Football b) Rugby League c) Rugby Union
d) Tennis e) Cycling f) Swimming
g) Athletics (Please indicate exact sport/s)
h) Others:

5. Please indicate the age group of the athletes you are working with. Please indicate all the applicable answers.

a) Junior b) Senior c) Development squad (U21/U23)

6. Please indicate from the list below your experience in your current support role.

a) <1 year b) 1–2 years c) 2–5 years
d) 5–10 years e) 10+ years

7. Please indicate the option that fits best your current role.

a) Part-time b) Full-time c) Self-employed

8. Please indicate from the list below your highest academic qualification.

a) Undergraduate degree
b) Postgraduate degree
c) Doctoral degree

9. Have you used / do you plan on using tart cherry supplements with your athletes? If your answer is 'd' please skip to Section 3. If you use(d) tart cherry supplementation, please indicate in which sport(s).

a) I currently recommend tart cherry supplements to athletes
b) I previously recommended tart cherry supplements to athletes but no longer do so
c) I have not recommended tart cherry supplements to athletes so far, but I plan on doing so in the future
d) I have never recommended tart cherry supplements to athletes and do not plan to do so

Sport(s):

Section 2. Use of tart cherry supplements as part of applied practice.

1. Who is the main initiator for conversations around tart cherry supplementation?

a) Athletes
b) Yourself
c) Other sports science staff members
d) Coaches
e) Others:

2. What was / is / will be your athletes' goal when using tart cherry supplements? Please indicate all the applicable answers.

a) Improving post-competition recovery
b) Improving sleep quality and duration
c) Acute supplementation to improve performance
d) Enhancing training adaptations
e) Improve immune function
f) Others:
g) Unsure

3. For which type of sports did you / do you / will you use acute supplementation (1-2h pre competition) with tart cherry? Please indicate all the applicable answers

a) Strength-based sports
b) Endurance-based sports
c) Power-based sports
d) Intermittent-running based sports
e) Others:
f) None
g) Unsure

4. What type of tart cherry supplement did you / do you / will you recommend as part of your practice? Please indicate all the applicable answers

a) Tart cherry concentrate
b) Tart cherry juice
c) Tart cherry powder
d) Other:

e) Unsure

5. During which periods did you / do you / will you use tart cherry supplements as part of your practice? Please indicate all appropriate answers

a) Before and After every match / competition
b) During periods with multiple condensed athletic events
c) During pre-season or demanding training blocks
d) Daily basis

6. What was / is / will be the usual duration of your supplementation protocol? Please indicate all appropriate answers

a) 2-3 days	b) 3-5 days	c) 5-8 days
d) continuous use	e) I only use acute tart cherry supplementation	
f) others	g) unsure	

7. What was is / will be the usual duration of your pre-loading supplementation protocol when using tart cherry supplements for enhancing recovery?

a) 1 day prior to competition
b) 2 days prior to competition
c) 3 days prior to competition
d) 4-5 days prior to competition
e) 6-8 days prior to competition
f) Unsure

8. What was / is / will be the quantity (mg/day) of polyphenols recommended as part of your supplementation protocol?

a) 300mg - 600mg/day
b) 600mg – 800mg/day
c) 1000mg – 1400 mg/day
d) Unsure
e) Other:

9. Do you / did you / will you use different quantities of polyphenols for acute and long-term tart cherry supplementation?

a) No
b) Yes (Please provide the dosages):

10. What is the main source you used for informing your supplementation protocol?

a) Relevant literature
b) Manufacturer recommendations
c) Consultations/Discussions with academic involved in tart cherry/polyphenols research
d) Other sports nutrition / strength and conditioning practitioners
e) Other sources:

Section 3. Attitudes towards tart cherry supplements

1. Do you believe that the current literature supports any of the following benefits for tart cherry supplementation? Please indicate all appropriate answers.

a) Improved post-exercise recovery
b) Improved sleep quality and duration

c) Improved endurance exercise performance
d) Improved repeated sprint performance
e) Improved strength performance
f) Enhanced training adaptations
g) Enhanced immunity and general health
h) Enhanced muscle mass development
i) Enhance injury rehabilitation
j) No effect
k) Others:

2. For which type of sports do you believe the literature supports the ergogenic effects of acute tart cherry supplementation? Please indicate all the applicable answers

a) Strength-based sports
b) Endurance-based sports
c) Power-based sports
d) Intermittent-running based sports
e) None
f) Others

3. Please indicate in a short sentence if you have noticed any improvements or received any positive feedback from your athletes following tart cherry supplementation

a) Sentence:
b) Does not apply to me as I have not used tart cherry supplements with my athletes

4. How do you believe tart cherry supplementation may affect training adaptations?

a) Likely to have a positive effect
b) Likely to have a negative effect
c) Likely to have no effect
d) Do not have an opinion on this

5. What is your biggest challenge when deciding on whether and how to develop your tart cherry supplementation protocol?

a) Current literature shows mixed findings regarding the efficacy of the supplement
b) Lack of clarity regarding the existence of an optimal supplementation protocol (duration and dose)
c) Lack of research illustrating the effects on training adaptations
d) Difficulties in choosing between available products on the sports supplements market
e) Supplement pricing
f) Prioritising other supplements (Please indicate which supplements):
g) Others (please indicate)

6. What feedback have you received from your athletes regarding the palatability of tart cherry supplements?

a) Positive, most athletes enjoy the taste
b) Negative, most athletes do not enjoy the taste
c) Neutral, the athletes take the supplement only because it benefits them
d) Does not apply to me as I have not used tart cherry supplements with my athletes

7. Did your athletes experience any side effects with tart cherry supplementation?

a) No

b) Yes (Please indicate the side effect)
c) Does not apply to me as I have not used tart cherry supplements with my athletes

8. What type of research would you like to see conducted in the future in this area? Please indicate all the applicable answers

a) Mechanistic studies illustrating the molecular pathways of action for tart cherry polyphenols
b) Applied studies looking at the effects of tart cherry supplementation on post-exercise recovery in elite athletes
c) Studies looking at the effects of tart cherry supplementation on sleep quality and duration in active individuals
d) Mechanistic studies looking at the effects of tart cherry supplementation on training adaptations
e) None
f) Other (Please indicate):

9. What type of tart cherry product would you like to see developed in the future? Please briefly describe why.

a) Beverage
b) Concentrate
c) Gel
d) Bar
e) Other
Reason:

Publisher's Note: MDPI stays neutral with regard to jurisdictional claims in published maps and institutional affiliations.

© 2020 by the authors. Submitted for possible open access publication under the terms and conditions of the Creative Commons Attribution (CC BY) license (http://creativecommons.org/licenses/by/4.0/).
